# Study on the Forming Process and Deformation Behavior of Inner Ring in the Wheel Hub Bearing Based on Riveting Assembly

**DOI:** 10.3390/ma12223785

**Published:** 2019-11-18

**Authors:** Wei Xiong, You Wang, Xiao-Ping Li, Song Mei, Zhu-Xin Tian

**Affiliations:** 1 School of Mechanical Engineering, Huazhong University of Science and Technology, Wuhan 430074, China; xwhubei@163.com; 2 School of Mechanical Engineering, Hubei University of Arts and Science, Xiangyang 441000, China; zhuxintian1987@sina.com; 3 Hubei New Torch Technology Co., Ltd, Xiangyang 441000, China

**Keywords:** Wheel hub bearing, Orbital forming, Forming process, Deformation behavior, Numerical simulation

## Abstract

The orbital riveting process has been successively adopted in the assembly of wheel hub bearing, due to its special merits of high efficiency, low cost, and so on. The forming process and deformation behavior of the inner ring have significant influence on the axial clamping force and bearing clearance, however, which haven’t been addressed yet. In this study, a numerical simulation platform for the assembly of the hub bearing is established by the joint use of the static implicit and dynamic explicit algorithms. Based on the platform, the deformation process and deformation behavior of the inner ring are investigated, along with the interference assembly and riveting assembly on the loading process of the inner ring. Finally, relevant experimental verifications are carried out to consolidate the simulation results. The research findings could be used to guide the design and optimization of the axial clamping force and bearing clearance.

## 1. Introduction

Orbital forming is a type of incremental metal forming process, which is mainly used in gear and automobile half-shaft processing due to its high efficiency, energy saving, low cost, and low noise [1,2,3,4,5]. In the past two decades, with the growing requirements of lightweight, integration, and reliability, the orbital riveting technology has been gradually applied to the manufacture of the automobile wheel hub bearings [6,7].

In orbital riveting process, the shaft end of the inner flange is flanged progressively with the rivet head pushing down, and finally fits closely with the upper end surface of the inner ring, so as to achieve the purpose of assembling and axial clamping. Currently, the machine manufacturers are mainly located in the United States, Japan, Germany, and Poland [8,9]. The research on orbital riveting process is mainly concentrated on the motion trajectory, inclination, and feed rate of the riveting head, the material and structure of the shaft end, and the lubrication state between the riveting head and the shaft end.

Toda et al. [10] outlined the riveting assembly process of hub bearing, and also carried out reliability analysis using failure mode and effects analysis (FMEA). The influence of the process parameters, such as the round corner of the inner ring, the fillet radius of the shaft end after riveting assembly, the pressure imposed by the riveting head, and the hardness of the shaft end on the axial clamping force were investigated experimentally. The axial principal stress distribution of the hub bearing was also obtained through finite element analysis (FEA). In their study, the authors pointed out that the performance of the hub bearing with riveting assembly is not lower than the case with bolt tightening.

Kajihara [11] elaborated the application of computer aided engineering (CAE) in strength, stiffness, riveting process, fretting, and weight reduction of hub bearing. Munshi et al. [12] studied the influence of feed rate and inclination angle of the riveting head on the maximum hoop stress and the deformation of inner ring. To reduce the computation time and volume change, Moon et al. [13] proposed an efficient rigid-plastic FEA method and algorithm, which had been successfully applied to the riveting process of hub bearing. Cho et al. [14] explored the riveting process by establishing 2D axisymmetric and 3D finite element models, and the results confirmed that the dynamic explicit method was suitable for the design and optimization of the riveting process. 

To save computation time and ensure the accuracy of solution, Nam et al. [15] investigated the assembly process of hub bearing using cyclic symmetry finite element model. The results revealed that the approach has a better balance between the calculation efficiency and the solution accuracy when the symmetry angle is equal to 60 degrees. Joshi et al. [16] conducted comparison of the hub inner shaft design by using ABAQUS Explicit module. 

Qu et al. [17,18] expounded on the riveting process with dynamic explicit algorithm. The process parameters of motion trajectory, stroke, and inclination angle of the riveting head, the friction coefficient at the interface between the riveting head and the shaft end on the assembly quality of hub bearing had been analyzed systematically. Moreover, to overcome the nonlinearity and instability of riveting process, an optimization strategy based on the surrogate model had been proposed for the optimization design of the riveting head, and the results demonstrated that the approach can be adopted to improve the service life of hub bearings [19]. Yang et al. [20] conducted theoretical analysis and experimental study of the riveting process, and focused on the effect of the motion trajectory, inclination angle, and duration time of riveting head on the axial clamping force. By taking a hub bearing case, they gave an optimization parameter combination and conducted relative experimental validation. Xiao et al. [21] studied the influence of the motion trajectory, feed rate, and riveting machine structure on the axial clamping force. 

The axial clamping force and bearing clearance are the critical technical specifications for hub bearing riveting process, however, which haven’t been claimed clearly in the past. In this study, the deformation behavior of the inner ring has been studied thoroughly, since the inner ring deformation has a direct influence on the bearing clearance and clamping force. In addition, the existing research achievements are carried out by using the dynamic explicit algorithm, without considering the processes of interference assembly and riveting unloading. Based on the background above, the present work carried out numerical simulation for the whole assembly processes of wheel hub bearing, by combining with the dynamic explicit and static implicit algorithms in the ABAQUS platform. Specially, the deformation behavior of the inner ring was studied thoroughly. Finally, experimental validations were conducted to consolidate the simulation results. The research findings can be used to guide the design and optimization of the axial clamping force and bearing clearance, which is the critical technology for hub wheel bearing manufacturing.

## 2. Experimental Platform

### 2.1. Riveting Assembly Process

**Figure 1:**
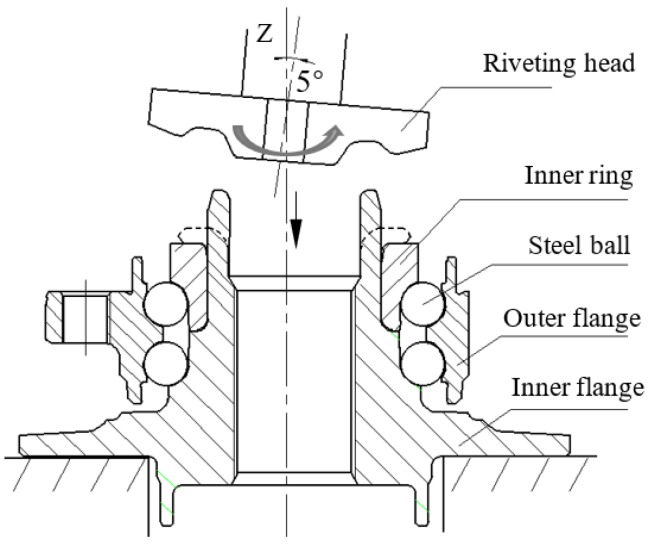
Schematic of riveting assembly process.

As shown in Figure 1, the riveting head is titled and rotated around the Z-axis during the riveting assembly process. The process can be divided into four stages. Firstly, the riveting head is rapidly pushed downward to establish contact with the shaft end. Secondly, the riveting head is continuously pushed downward until the shaft end is completely in contact with the upper end surface of the inner ring. The third stage is also called the smoothing stage, during which the vertical displacement of the riveting head remains constant for a while to flatten the shaft end. Finally, the inner ring and the inner flange are undergoing unloading process, with the riveting head lifting up.

### 2.2. Forming Apparatus

**Figure 2:**
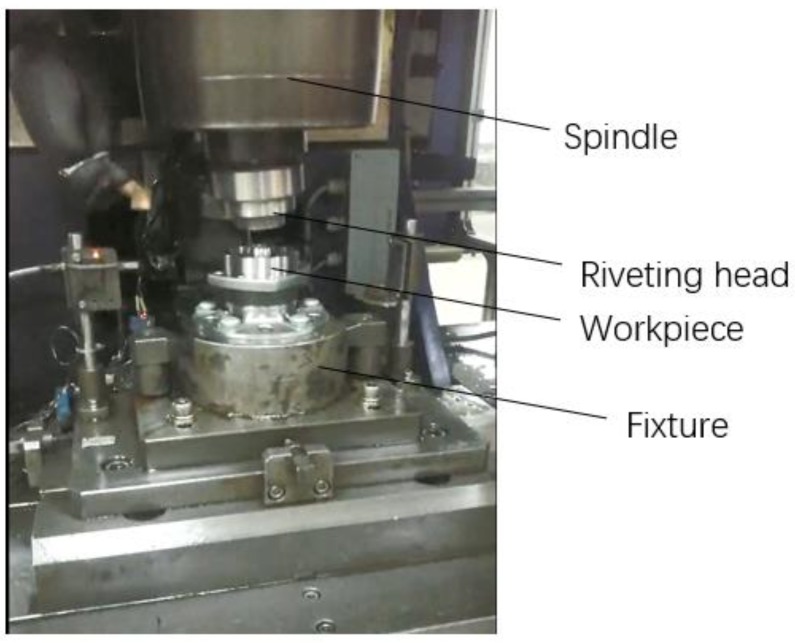
Riveting machine.

The forming apparatus used in the study was manufactured by SCFA Company, Chilgok-gun, South Korea, as shown in Figure 2. The apparatus is driven by electromagnetic force, and the maximum vertical riveting force is 300 kN. During the riveting assembly process, the displacement of the riveting head is controlled and detected by a grating sensor. The hub bearing unit is provided by NTP Company, Xiangyang, China.

**Figure 3:**
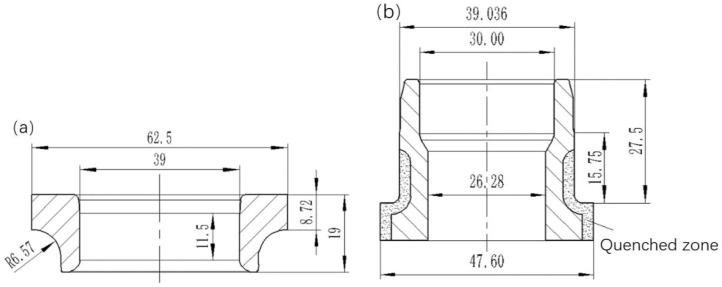
Key parts. (**a**) Inner ring. (**b**) Inner flange.

The steel balls contacted with the races and the outer flange have little effect on the deformation of inner ring, which can be neglected for the experiment and simulation. Before riveting process, the inner ring and inner flange are assembled with interference fit (0.036 mm). The key parts and their main dimensions are shown in Figure 3.

### 2.3. Experimental Tests

The vertical riveting force is measured through a built-in pressure sensor, and the curve of the vertical riveting force versus the vertical displacement of the riveting head is printed after riveting assembly process.

**Figure 4:**
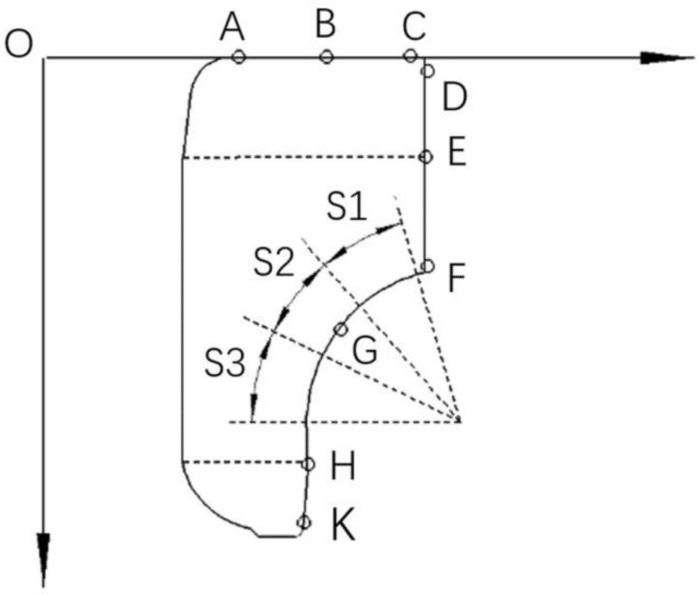
Measurement points along the generatrices of the inner ring.

The radial displacements of the inner ring are measured by a digital universal length meter with the accuracy of 0.001 mm, and the measured points along the side generatrix are marked with D, E, F, G, H, and K, as shown in Figure 4. The vertical displacements are measured by a dial indicator provided by Ningbo Instrument & Meter Co., Ltd, Ningbo, China with the accuracy of 0.001 mm, and the measured points along the upper generatrix are marked with A, B, and C. It is noted that all the measurements of displacement are conducted after the interference/riveting assembly process. 

## 3. Finite Element Modeling and Numerical Simulation Analysis of the Riveting Assembly

### 3.1. Finite Element Modeling

Based on the general finite element software ABAQUS provided by Dassault Systèmes Simulia Corp., Providence, RI, USA, numerical simulation platform (NSP) of the hub bearing assembly was established by the joint use of static implicit and dynamic explicit algorithms. The numerical simulation of interference assembly and riveting assembly processes have been carried out based on the NSP. Specific steps are as follows: Firstly, the interference assembly was carried out by using ABAQUS Standard. Secondly, the riveting loading process was simulated with ABAQUS Explicit by taking the simulation result above as the predefined stress field. Finally, the springback analysis was conducted in ABAQUS Standard by taking the simulation result of the previous step as the predefined stress field.

**Figure 5:**
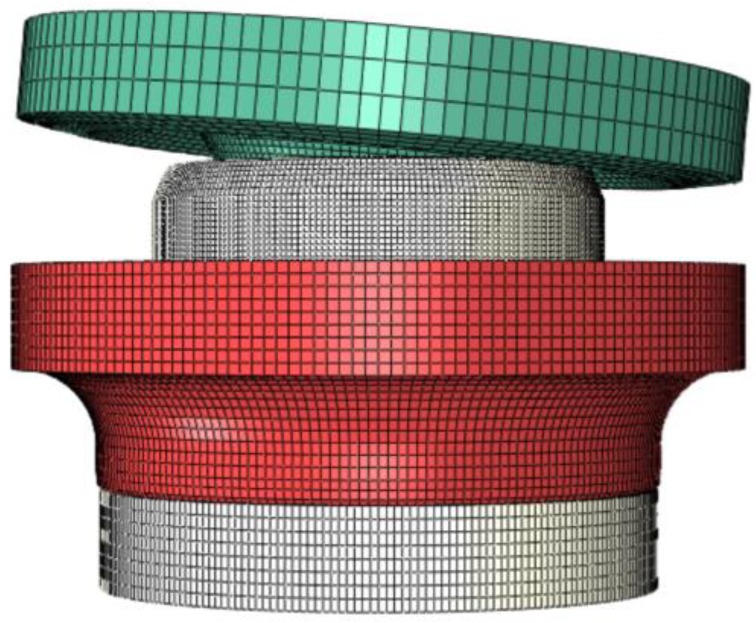
Finite element model of hub bearing assembly.

The lower part of the inner flange has little effect on the riveting process, which can be neglected for simulation. The FEM of hub bearing assembly is shown in Figure 5, where the linear reduction integration element C3D8R was adopted to mesh the inner flange, inner ring, and riveting head, and then the riveting head was transformed into a rigid part. The friction forces at the interfaces were assumed to follow Coulomb’s model, and the friction coefficients were set to be 0.15.

**Figure 6:**
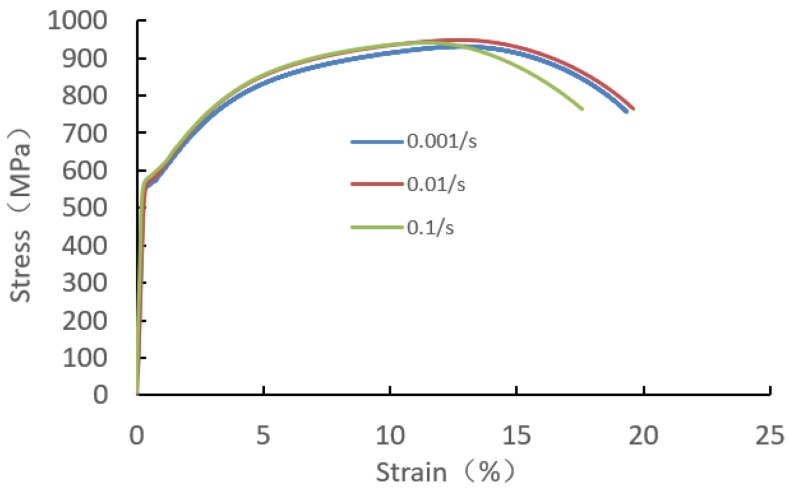
Real stress–strain curve of quenched and tempered 65Mn.

The material of inner ring is quenched GCr15 bearing steel. The base material of inner flange is quenched and tempered 65Mn, and the quenched area is shown in Figure 3. For the material of quenched and tempered 65Mn, the real stress-strain curves under different strain rates were measured by using SHT4305 Microcomputer Universal Test Machine as shown in Figure 6. As the strain-stress curves are close to each other before necking, the average stress values are adopted to model the plastic strain–stress relationship. The mechanical behavior of the material is assumed as an isotropic, elastic-plastic model with isotropic hardening law and von Mises yield criterion. 

**Figure 7:**
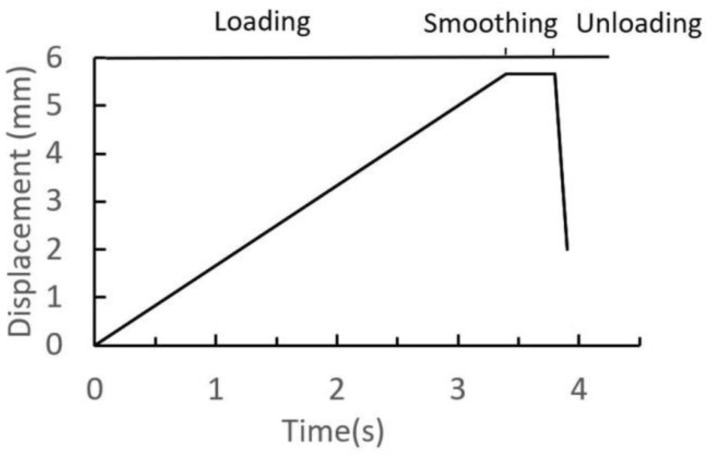
Time-displacement curve of the riveting head.

Furthermore, the relative mechanical properties of quenched 65Mn and GCr15 are listed in Table 1, and both materials are modeled with isotropic, linear elastic behavior. The time-displacement curve of riveting head is given in Figure 7, in which the stages of the riveting assembly are depicted. Moreover, the rotational speed of riveting head is 600 r/min, and the speed remains constant during riveting loading and smoothing stages.

In order to save computation time, the mass scaling strategy was adopted. The reliability of the simulation results can be evaluated through the ratio of kinetic energy to internal energy. Generally, the simulation results are considered reliable when the ratio of is less than 5–10%.

### 3.2. Numerical Simulation of the Riveting Loading Process

**Figure 8:**
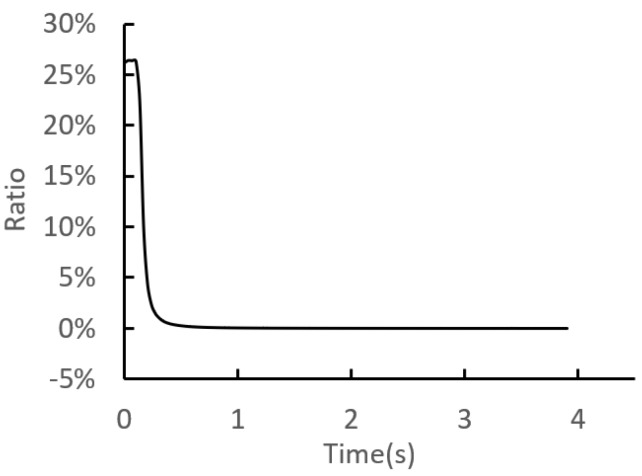
The ratio of kinetic energy to internal energy during the riveting loading process.

The ratio of kinetic energy to internal energy is illustrated in Figure 8. It can be found that the ratio is less than 1% during the loading process, indicating the simulation results are smooth and reliable.

**Figure 9:**
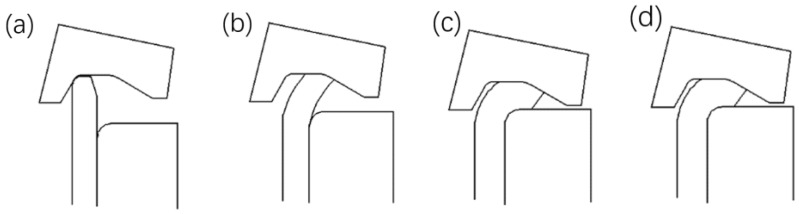
Representative moments during the riveting loading process. (**a**) 0.5 s. (**b**) 2.5 s. (**c**) 3.4 s. (**d**) 3.8 s.

**Figure 10:**
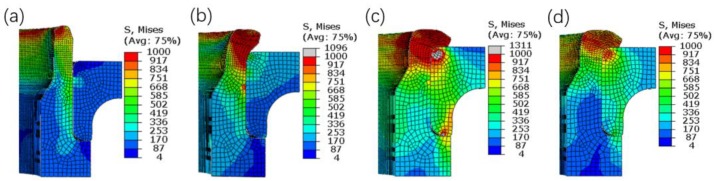
Equivalent stress contour plots at the representative moments. (**a**) 0.5 s. (**b**) 2.5 s. (**c**) 3.4 s. (**d**) 3.8 s.

**Figure 11:**
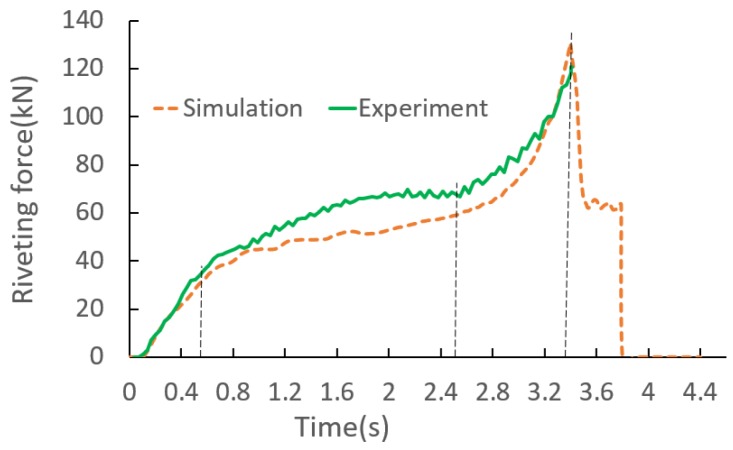
Vertical riveting force in the process of riveting assembly.

In riveting assembly process, several representative contact moments between the riveting head, inner flange, and inner ring are selected and shown schematically in Figure 9, among which, 0.5 s is the stable contact moment between the riveting head and the shaft end, 2.5 s is the initial contact moment between the round corner and the shaft end, 3.4 s is the moment of the riveting head locating at the lowest position, and finally 3.8 s is the end moment of the smoothing stage. The equivalent stress contour plots at the representative moments are shown in Figure 10, and the vertical riveting force versus time is given in Figure 11.

By combining with Figure 10 and Figure 11, the changing process of the vertical riveting force is explored, along with the loading process of the inner ring. The time period from zero to 0.5 s is the lead-in stage of contact between the riveting head and the shaft end, during which the vertical riveting force is increased with the loading displacement increased. In this stage, the deformation of the inner ring is imposed by the radial swelling of the interference surfaces, and the main interaction region is located below the round corner of the inner ring. The period 0.5–2.5 s is the stable flanging stage, during which the increasing trend of the vertical riveting force becomes slower, and the main interaction region moves upward. In the period from 2.5 to 3.4 s, the shaft end is contacted with the round corner of the inner ring gradually. During the period, the vertical riveting force is increased sharply, and the main interaction region moves to the area around the corner of the inner ring. Meanwhile, a strong local interaction area appears at the bottom mating surface of the inner flange and the inner ring. Finally, the period from 3.4 to 3.8 s refers to the smoothing stage of the riveting assembly. In this stage, the position of the main interaction region is almost the same of that in the previous period. Furthermore, it should be noted that the smoothing stage here is adopted to flatten the upper end surface of the flange, and the vertical riveting force is decreased sharply as the deformation resistance of the shaft end reduced. It is noted here that, at the main interaction region, the equivalent stress values of the inner ring are increased with the loading displacement of riveting head increased, but which are decreased in the smoothing stage of riveting assembly.

The experimental curve of the vertical riveting force versus time is given in Figure 11. The comparison of simulation and experimental results demonstrate that the changing trends are the same, i.e., the vertical riveting force is increased with the time (displacement) increased in the time period 0–3.4 s. Meanwhile, the maximum simulated and experimental values are 131.2 kN and 119.7 kN, respectively, and the relative error between them is 8.76%. The results demonstrate that the simulation results are in good agreement with the experimental data.

## 4. Results and Discussion

### 4.1. The Influence of the Interference Assembly

**Figure 12:**
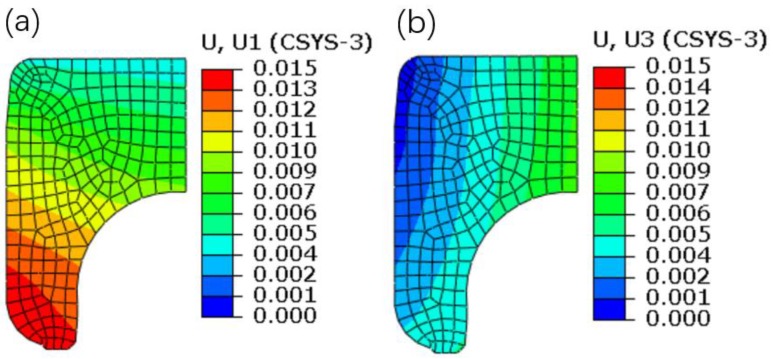
Displacement contour plots of the inner ring. (**a**) Radial displacement. (**b**) Axial displacement.

The displacement contour plots of the inner ring are shown in Figure 12. It can be seen that the radial displacement is increased from top to bottom, showing an increasing trend with the decrease of the inner ring thickness. On the other hand, the axial displacement is increased from inside to outside, which is due to the restriction of extrusion and friction of the interference surface. It should be pointed out that the values of the radial and axial displacements are positive, which demonstrates that the interference assembly not only enlarges the inner ring radially, but also elongates the inner ring axially. 

**Figure 13:**
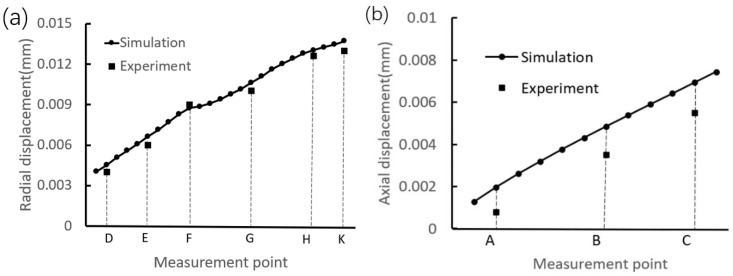
Displacement distributions. (**a**) Along the side generatrix. (**b**) Along the upper generatrix.

To quantitatively analyze the interference assembly on the deformation of the inner ring, Figure 13 shows the displacement distributions along the upper and side generatrices. It can be observed that the changing trend of the simulated radial displacement is the same as that of the measured displacements, that is, the radial displacement is increased along the direction from the marked point D to point K. In addition, it is found that, for the radial displacement, the maximum absolute error between the simulated and experimental values is less than 0.001 mm; and for the axial displacements of the marked points A, B, and C, the maximum absolute error is slightly greater than 0.001 mm. The above analyses indicate that the simulated data are in good agreement with the measured data.

It is known that the changes of displacement and radius have significant influence on the bearing clearance, as the steel balls are in close contact with the raceway of the inner ring. Therefore, it is necessary to explore the influence of the interference assembly on the radius change of the arc segment. The least square method is adopted to fit the curvature radii of the separated arc segments, and the fitting results are listed in Table 2. It is found that the interference assembly has little effect on the radius change of the arc segment. Therefore, the interference assembly on the radius change can be neglected in precision design of bearing clearance.

### 4.2. The Influence of the Riveting Loading Process

**Figure 14:**
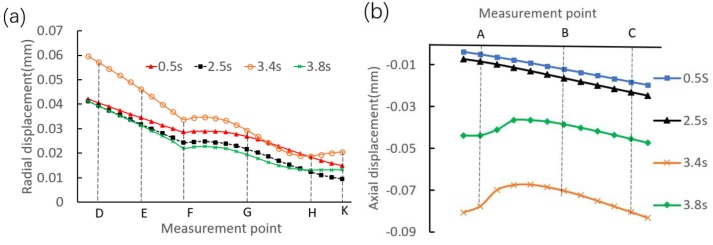
Displacement distributions of the inner ring at the representative moments. (**a**) Along the side generatrix. (**b**) Along the upper generatrix.

During the riveting loading process, the riveting head imposes periodic contact pressure on the shaft end. This section mainly conducts deformation trend analysis, as the inner ring deformation involves obvious contact, force, and structural nonlinearity. The displacement distribution curves along the generatrices are given in Figure 14. Here it should be noted that the selected generatrices are located right below the contact region between the riveting head and the shaft end.

For the radial displacement, the changing trends are almost the same from an overall perspective, i.e., the radial displacement at each representative moment shows a decreasing trend along the side generatrix. On the other hand, the variations of the axial displacement at each representative moment are all small, and the absolute axial displacement is increased from the marked point A to point C from an overall perspective. It is also found that the axial displacement shows a nonlinear distribution at the moments of 3.4 s and 3.8 s. This is because the strong local pressure occurs at the contact region between the flange and the inner ring. In addition, it can be seen that at the moments of 3.4 s and 3.8 s, the axial displacement shows an approximately linear distribution in the region away from point A. 

**Figure 15:**
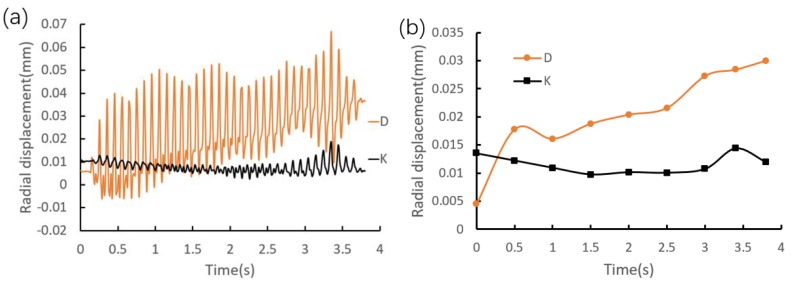
Radial displacement distributions at point D and point K. (**a**) Global change. (**b**) Fitting value change.

By taking point D and point K as examples, the quantitative analyses of the riveting loading process on the radial displacement are investigated, and the distribution curves are shown in Figure 15a. It can be seen that the radial displacement shows a strong periodic trend with a period of 0.1 s, which is the same as the rotation cycle of the riveting head. In order to measure the radial displacement during the riveting loading process, the least square method is adopted to perform circle fitting, and the results are transformed and illustrated in Figure 15b. The figure illustrates that the radial displacement at point D is increased from an overall view, and the radial variation is 0.026 mm. However, the radial variation is 0.002 mm at point K, because the point K is far away from the local contact region.

**Figure 16:**
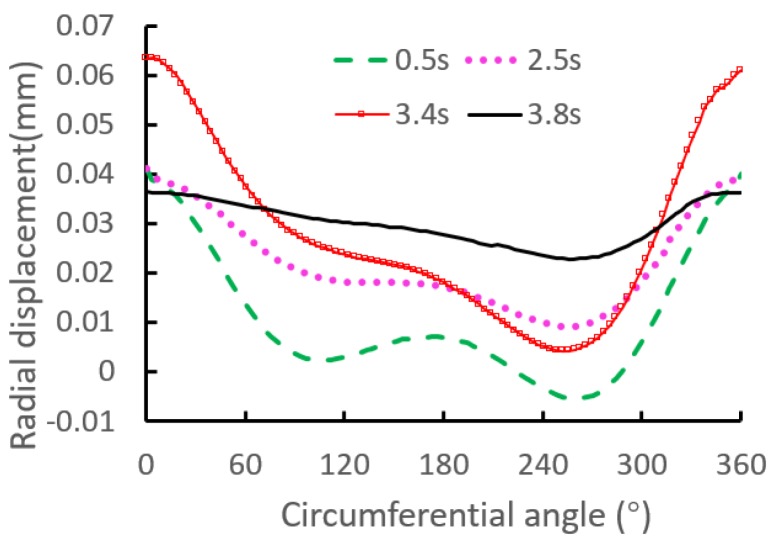
Periodic trend of the axial displacement at point D.

Furthermore, to visualize the periodic trend of the radial displacement, the relationship of the radial displacement versus circumferential angle is shown in Figure 16. The figure indicates that the displacement shows an irregular distribution in the circumferential direction, and the maximum displacement is located below the local contact region. The reason of the above phenomena is that the displacements at the rest of circumferential angles are restricted by the extrusion and restriction of the interference surface.

**Figure 17:**
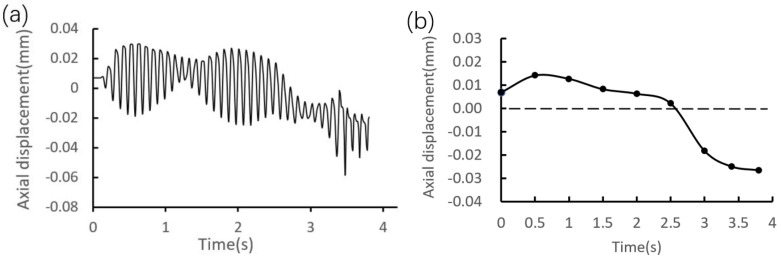
Axial displacement distribution at point C. (**a**) Global change. (**b**) Average value change.

On the other hand, by taking point C as an example, the quantitative analyses on the axial deformation are studied, and the distribution curve is given in Figure 17a. It is found that the cycle period of the axial displacement is the same as the rotation cycle of the riveting head. In order to quantitatively measure the riveting loading process on the axial displacement, the average displacements along the circumferential points passing through point C are calculated and illustrated in Figure 17b. It can be seen that the average displacement shows a decreasing trend from an overall view. Here it should be stated that, before the shaft end contacts with the round corner, the deformation of the inner ring is imposed by the radial swelling of the interference surface, which results in axial elongation of the inner ring. However, after the shaft end contacted with the round corner, the deformation of the inner ring is exerted mainly through the pressure imposed by the flange, which results in axial compression of the inner ring. 

**Figure 18:**
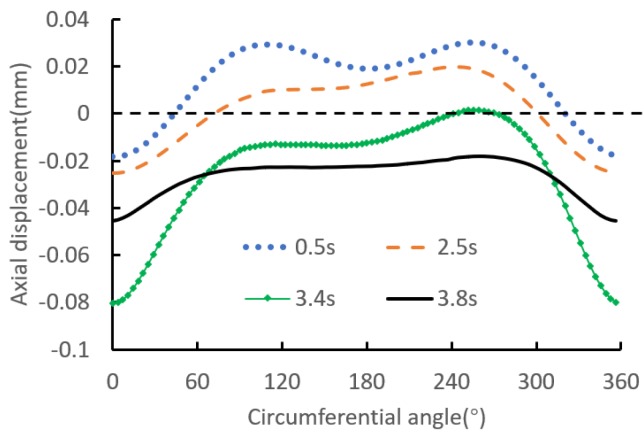
Periodic trend of the axial displacement at point C.

Furthermore, in order to visualize the periodic trend of the axial displacement, Figure 18 shows the relationship of the axial displacement versus circumferential angle. It can be seen that in the process of riveting loading, the axial displacement shows an irregular deformation trend, and the minimum displacement is located at the local contact region. The reason is that the displacements at the rest of circumferential angles are generated in unconstrained state.

### 4.3. The Influence of the Riveting Unloading Process

**Figure 19:**
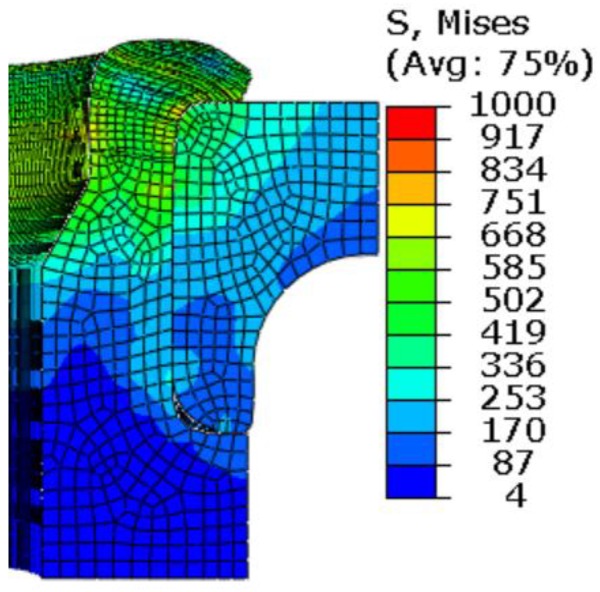
Equivalent stress contour plot after springback.

**Figure 20:**
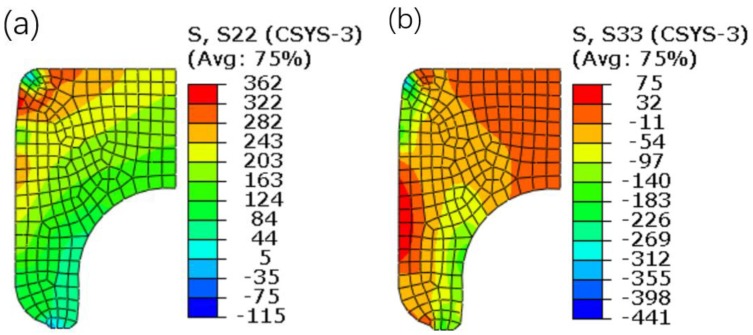
Stress contour plots of the inner ring. (**a**) Circumferential stress. (**b**) Axial stress.

The equivalent stress contour plots after springback are shown in Figure 19. It is found that the values of equivalent stress are decreased, compared with the equivalent stress distribution shown in Figure 10d. It is known that the axial clamping force is generated at the contact region between the flange and the upper surface of the inner ring. Therefore, it can be speculated that the residual stress of the inner ring has significant influence on the axial clamping force. Figure 20 shows the stress contour plots of the inner ring. The figure indicates that the distribution of the circumferential stress is more uniform, and their values are much larger. Therefore, it can be preliminarily determined that the circumferential stress is more significant in affecting the axial clamping force. However, the formation mechanism of the axial clamping force will not be further discussed in the study.

**Figure 21:**
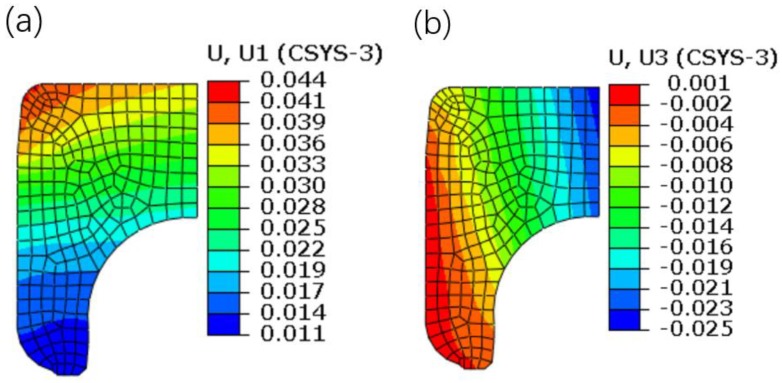
Displacement contour plots of the inner ring. (**a**) Radial displacement. (**b**) Axial displacement.

**Figure 22:**
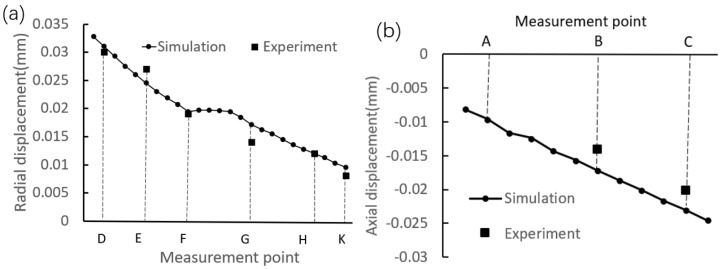
Displacement distributions along the generatrices. (a) Along the side generatrix. (b) Along the upper generatrix.

The displacement contour plots of the inner ring are shown in Figure 21. As a whole, the radial displacement is decreased gradually from top to bottom, and the maximum displacement is located at the area near the round corner. On the other hand, the axial displacement is increased gradually from inside to outside, and the maximum displacement is located at the point of intersection between the side generatrix and the upper generatrix. In order to quantitatively analyze the deformation of the inner ring, the curves of displacement distribution are given in Figure 22. It can be seen that the radial displacement is decreased linearly along line DF and line GK, and the variation at the arc segment FG is small. In addition, it is found that the axial displacement is decreased linearly from the inside to the outside. Meanwhile, by comparing with Figure 14b, it is found that the axial deformation near point A is almost totally recovered.

Radial and axial displacement measurements were carried out, and the experimental values were compared with the simulated ones. The results demonstrate that the change trend of the simulated displacements is the same as that of the experimental displacements. Furthermore, it is found that for the radial displacement, the maximum error is at point G and the absolute error value is 0.002 mm, and for the axial displacement, the maximum error is at point B and the absolute error value is 0.003 mm. Therefore, it can be concluded that the simulation results are in good agreement with the experimental results.

As mentioned above, the changes of displacement and radius have significant influence on bearing clearance. Therefore, the curvature radii of the separated arc segments are fitted by using the least square method, and the results are listed in Table 3. It can be seen that the maximum absolute error is 0.026 mm. However, the contact region between the steel balls and the raceway is located at a very small area at the arc segment S2. Therefore, the attention should be paid on the displacement change instead of the radius change in precision design of the bearing clearance.

**Figure 23:**
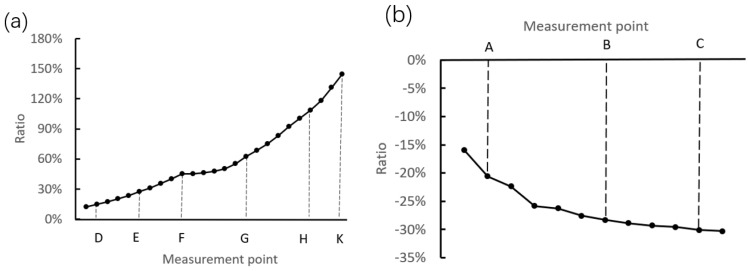
Displacement ratio of the interference assembly to the riveting assembly. (**a**) Along the side generatrix. (**b**) Along the upper generatrix.

By combining with Figure 13 and Figure 22, the curves of displacement ratio are drawn as shown in Figure 23. Here, the displacement ratio is defined as the value of the displacement after interference assembly to the displacement after riveting assembly. It can be seen that the ratio is increased along the upper generatrix, which indicates that the influence of the interference assembly is increased gradually. In addition, it should be pointed out that the radial displacement ratios are larger than 40% in the arc segment, which indicates that the interference assembly induces large deformation at the raceway. Therefore, the influence of the interference assembly should be considered in the precise control of bearing clearance. On the other side, the absolute value of the axial displacement ratio is increased slowly along the side generatrix (smaller than 30%), which indicates that the interference assembly has a smaller effect on the axial displacement. 

**Figure 24:**
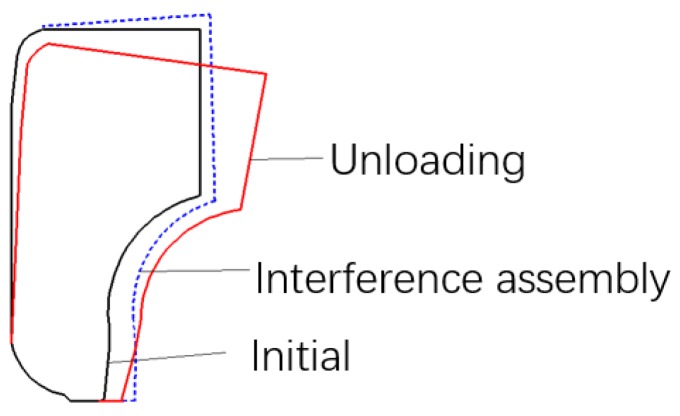
Changing process of the inner ring section (deformation scale × 100).

Moreover, in order to visually show the influence of the interference and riveting assemblies on the deformation of the inner ring, the changing process of the cross-section of the inner ring is illustrated in Figure 24. It can be seen that the interference assembly results in a slight upward angle, while the interference assembly leads to a small downward angle. This conclusion is of guiding significance in bearing clearance design, and in section optimization of the inner ring. 

## 5. Conclusions

Based on the numerical simulation platform, the study explored the deformation process and behavior of the inner ring during the whole assembly processes. The changing trends of the radial and axial displacements are analyzed systematically. Moreover, the relevant experimental tests were carried out to consolidate the simulation results. The main conclusions are as follows:

(1) The interference assembly enlarges the inner ring radially and elongates the inner ring axially, while the riveting assembly enlarges the inner ring radially and compresses the inner ring axially. In addition, it is found that the interference assembly results in a slight upward angle, while the riveting assembly leads to a small downward angle. The conclusion is of guiding significance in bearing clearance design and in section optimization of the inner ring.

(2) The riveting assembly has greater influence on the deformation of the inner ring, compared with the interference assembly as a whole. However, the influence of the interference assembly should be considered when performing precise clearance design, as the ratio of the radial displacement is larger than 40% in the arc segment of the raceway.

(3) Both the interference assembly and the riveting assembly are having little influence on the radius change at S1 and S2 segments. Therefore, when performing precise clearance control, the main attention should be focused on the deformation, rather than the radius change.

(4) In the early stage of the riveting loading, the deformation of the inner ring is exerted through the swelling of the interference surface. As the riveting head moves down, the main interaction region between the inner ring and the inner flange moves up and gradually moves to the region near the round corner of the inner ring. Later, the deformation of the inner ring is mainly exerted through the pressure imposed by the flange. After springback, the values of the circumferential stress are much higher than those of the axial stress from an overall point of view, which is likely to have a larger influence on the axial clamping force.

(5) During the riveting loading process, the radial and axial displacements are showing periodic irregular change, and the cycle time is the same as that of the riveting head rotation. Moreover, the radial displacements in the circumferential direction are the same as the axial displacements, and the strong local axial deformation has completely recovered after springback.

## Figures and Tables

**Table 1:** Mechanical properties of the quenched 65Mn and GCr15.

Mechanical Property	Density (kg/m^3^)	Young’s Modulus (GPa)	Poisson’ s Ratio	Yield Strength (MPa)
65Mn	7800	212	0.288	1400
GCr15	7800	220	0.3	1700

**Table 2:** Curvature radii at the separated arc segments after interference assembly.

Section	S1	S2	S3
Initial radius (mm)	6.57	6.57	6.57
After assembly (mm)	6.564	6.572	6.569
Absolute error (mm)	0.006	0.002	0.001

**Table 3:** Curvature radii of the separated arc segments after springback.

Section	S1	S2	S3
Initial radius (mm)	6.57	6.57	6.57
After unloading (mm)	6.574	6.564	6.544
Absolute error (mm)	0.004	0.006	0.026

## References

[1] Han X.H., Hua L. (2009). Comparison between cold rotary forging and conventional forging. J. Mech. Sci. Technol..

[2] Hildenbrand P., Lechner M., Vogel M., Herrmann H., Merklein M. (2018). Orbital forming of tailored blanks with two-sided local material thickening. Int. J. Adv. Manuf. Technol..

[3] Han X.H., Hua L. (2012). Investigation on contact parameters in cold rotary forging using a 3D Fe method. Int. J. Adv. Manuf. Technol..

[4] Eshtayeh M., Hrairi M. (2016). Recent and future development of the application of finite element analysis in clinching process. Int. J. Adv. Manuf. Technol..

[5] Nowak J., Madej L., Ziolkiewicz S., Plewinski A., Grosman F., Pietryzyk M. (2008). Recent development in orbital forging technology. Int. J. Mater. Form..

[6] Ishida H., Kaneko T. (2000). Development of hub unit bearings with swaging. NSK Tech. J..

[7] Numata T. (2005). Latest technical trends regarding hub unit bearings. Koyo Eng. J. Engl. Ed..

[8] Shivpuri R. (2013). Past developments and future trends in the rotary or orbital forging process. J. Mater. Eng. Perform..

[9] Zhou Z.X., Xiao Y.Y., Li W. (2014). Study of a new machine tool for the riveting and assembly of automotive hub bearing unit. J. Hunan Univ. Natl. Sci..

[10] Toda K., Ishii T., Kashiwagi S., Mitarai T. (2001). Development of hub units with shaft clinching for automotive wheel bearings. Koyo Eng. J. Engl. Ed..

[11] Kajihara K. (2005). Improvement of simulation technology for analysis of hub unit bearing. Koyo Eng. J. Engl. Ed..

[12] Munshi M., Shah K., Cho H., Altan T. Finite element analysis of orbital forming used in spindle/inner ring assembly. Proceedings of the 8th International Conference on Technology of Plasticity.

[13] Moon H.K., Lee M.C., Joun M.S. (2007). An approximate efficient finite element approach to simulating a rotary forming process and its application to a wheel-bearing assembly. Finite Elem. Anal. Des..

[14] Cho H.J., Koo J.S. (2008). Orbital forming simulation of automotive hub bearing using the explicit finite element method. Int. J. Mod. Phys. B.

[15] Nam C.H., Lee M.C., Eom J.G., Choi M.H., Joun M.S. (2014). Finite element analysis model of rotary forging for assembling wheel hub bearing assembly. Procedia Eng..

[16] Joshi D., Singh A.P. (2016). Comparison of hub inner shaft design options for orbital forming process by finite element method and subsequently effect on preload of generation-3 wheel hub bearing. IOSR J. Mech. Civ. Eng..

[17] Qu J., Zhang G.J. (2016). Numerical and experimental investigations of the shaft-clinching assembly process of automobile wheel-hub-bearing units. Proc. Inst. Mech. Eng. Part D J. Automob. Eng..

[18] Qu J., Zhang G.J. (2016). Determination of motion equation of rivet head during shaft riveting assembly process for wheel hub bearing units. J. Manuf. Sci. Eng..

[19] Qu J., Zhang G.J., Xu X.Q. (2016). Optimization design of rivet head for shaft riveting assembly of hub bearing unit. J. South China Univ. Technol. Natl. Sci. Ed..

[20] Yang J., Yang Y.S., Li W., Xiao Y.Y., Zhou Z.X. (2017). Theoretical analysis and experimental studies for riveting and assembly processes of automotive hub bearing units. China Mech. Eng..

[21] Xiao Y.Y., Zhou Z.X., Li W., Meng G.F. (2013). Axial force test and analysis in riveting assembly of automotive hub bearing unit. Appl. Mech. Mater..

